# Cross subject emotion identification from multichannel EEG sub-bands using Tsallis entropy feature and KNN classifier

**DOI:** 10.1186/s40708-024-00220-3

**Published:** 2024-03-05

**Authors:** Pragati Patel, Sivarenjani Balasubramanian, Ramesh Naidu Annavarapu

**Affiliations:** Department of Physics, Pondicherry University, Puducherry, 605014 India

**Keywords:** EEG signal, Emotion identification, Brain region, EEG channel selection, Tsallis entropy, Feature engineering, SEED data set, KNN classifier

## Abstract

Human emotion recognition remains a challenging and prominent issue, situated at the convergence of diverse fields, such as brain–computer interfaces, neuroscience, and psychology. This study utilizes an EEG data set for investigating human emotion, presenting novel findings and a refined approach for EEG-based emotion detection. Tsallis entropy features, computed for q values of 2, 3, and 4, are extracted from signal bands, including theta-θ (4–7 Hz), alpha-α (8–15 Hz), beta-β (16–31 Hz), gamma-γ (32–55 Hz), and the overall frequency range (0–75 Hz). These Tsallis entropy features are employed to train and test a KNN classifier, aiming for accurate identification of two emotional states: positive and negative. In this study, the best average accuracy of 79% and an *F*-score of 0.81 were achieved in the gamma frequency range for the Tsallis parameter *q* = 3. In addition, the highest accuracy and *F*-score of 84% and 0.87 were observed. Notably, superior performance was noted in the anterior and left hemispheres compared to the posterior and right hemispheres in the context of emotion studies. The findings show that the proposed method exhibits enhanced performance, making it a highly competitive alternative to existing techniques. Furthermore, we identify and discuss the shortcomings of the proposed approach, offering valuable insights into potential avenues for improvements.

## Highlights

- Subject independent human emotion identification is studied using SEED data set.
- Tsallis entropy is employed as feature and performance variation with Tsallis parameter (*q* = *2, 3, 4)* is examined.
- Performance of kNN classifier is examined with Tsallis entropy feature.
- Emotion identification at various levels is studied, brain region, EEG rhythms, brain hemisphere.
- Prospects of TsEn-based real-time emotion recognition framework is canvassed.

## Introduction

### Emotions and emotions recognition

The human brain is one of the most evolved brains among all other living organisms. Emotions result from cognitive mechanisms within billions of neurons subjected to situations and surroundings [1]. With the advancement of science and technology, the practice to explore and quantify the subjective emotions of human beings has gained little success but gaining higher accuracy remains challenging. The perception, situational reaction, and critical thinking biased with human emotions have been tedious to generalize or functionalize. However, a deeper and more accurate understanding of human emotion would essentially push forward the mental health care disciplines and next-generation artificial intelligence.

As a primitive categorization, human emotions were classified based on two major models, namely discrete emotion models, which rank emotions into happiness, sadness, anger, fear, and disgust [2]. The second model gives a preliminary qualification based on the emotion’s arousal and valance [3]. The model is called the bidimensional emotion model, which categorizes emotions in a two-dimensional plane within the four quadrants. The intensity of a particular emotion is quantified as its valance. The *x*-axis of the plane describes the valance of emotions. Distributions towards the left of the origin shows negativeness while towards the right indicates positiveness.

Similarly, the arousal axis grades the activation and calmness of the emotion from top to bottom. Systematic and scientific emotion detection is inevitable for generalizing and categorizing emotion and extending it to multidisciplinary science and technology development. This study is conducted on the SEED data set which is based on the discrete emotion model. However, these enormous data inevitably require an efficient classification to make reliable conclusions.

### Why EEG?

Technology has significantly evolved to detect human emotions through various methods. Signals from speech, physical posture, body language, and facial expressions are a few of them [4–6]. A more precise and clinical detection mechanism of human emotion has been established by Hans Berger et al. using electroencephalography (EEG) [7]. EEG involves the direct measurement of electric signals and their variations during brain activity that helps to digitalize subjective human emotions in the best way possible. The preference for EEG recordings over different alternatives is also substantiated by the fact that primary impulse in response to any input is generated in the brain. This impulse then subsequently transmits through the central nervous system to the rest of the peripheral systems. From this context, EEG recordings give the source's emotional response, whereas other physiological factors might be seen as a by-product of the brain’s response to the stimulus [8]. Thus, a substantial rise in the number of studies that employed EEG time series to build an emotion identification framework can be seen recently.

### Entropy-based emotion recognition

In the initial days of the EEG-based research, the EEG recordings were analyzed using linear methods, particularly in the frequency zone. However, it would be incorrect to characterize brain activity as linear. Neurons connect diversely and nonlinearly at all levels, cellular or global. Therefore, use of linear techniques alone will not provide a comprehensive account of brain’s electrical responses. Given this information, nonlinear algorithms have been employed further to unravel underlying information that remains undiscovered with classic linear approaches. Nonlinear approaches have consistently outscored the findings of linear algorithms in studying mental activities, including emotion identifications [9–11].

Entropy measures are being broadly used to build emotion recognition frameworks among the several nonlinear approaches found in the literature. Entropy, which describes the nonlinear properties of a nonstationary system, is the rate of information that a time series report. As a result, entropy measures are valuable tools for evaluating the chaotic dynamics of nonstationary systems like the brain. Several research studies have used these nonlinear approaches to extract emotional states from EEG records [12–14].

The concept of entropy first came in the field of thermodynamics. Further, it was adapted and redefined in the information theory by Shannon and known as Shannon’s entropy. Shannon entropy in signal analysis defines the amount of information a signal provides, indicating its complexity, irregularity, or unpredictability [15]. Later many entropy generalizations were formulated and effectively employed for various EEG-based medical research, including mental illnesses, epilepsy [16–18], Alzheimer’s [19–21], autism, and depression [22, 23], among others. Considering these outcomes, entropy measures were employed in studying emotion recognition from EEG signals [24–27].

### Previous entropy-based emotion studies

Literature shows that various entropy functions have been derived and used for emotion recognition with the EEG data set [13]. Table 1 summarises all the entropy works and our results from Tsallis entropy for emotion recognition. For simplification, all the entropies studied to date can be categorized as (i) regularity-based entropy (ii) Predictability-based or symbolic entropy, and the (iii) multiscale entropy.

**Table 1:** Comparison between the performance of existing entropy indices and the method proposed in the present study

Entropy indices	Database (No. of channels)	No. of subjects (Stimuli)	Emotions	Classifier	Accuracy	Reference (Year)
*Regularity-based entropy indices*						
Approximate entropy + others	Private (31 channels)	44 (Images)	HVLA, LVLA, LVHA	SVM	75.5%	[51] 2017
Quadratic sample entropy	DEAP (32 channels)	32 (Video)	Calm, distress	DT	75.29%	[52] 2016
Dynamic sample entropy	SEED (62 channels)	15 (Video)	Positive, negative	SVM	84.67%	[53] 2021
Clustering coefficient entropy	SEED (62 channels)	15 (Video)	Positive, negative	SVM	68.44%	[54] 2021
*Predictability-based entropy indices*						
Permutation entropy, AAPE, quadratic sample entropy	DEAP (32 channels)	32 subjects (Video)	Calm, negative stress	SVM	81.31%	[28] 2017
Spectral entropy, shannon entropy	DEAP (32 channels)	32 subjects (Video)	Valence and arousal,	LSSVM, D-RFE	78.96% (arousal) 71.43% (valence)	[29] 2017
Renyi entropy + others	DEAP (32 channels)	32 subjects (Video)	2, 3, 4 and 5 emotions	SVM	73.8–86.2%	[30] 2018
Kolmogorov entropy, shannon entropy, power-spectral entropy	Private (3 channels)	213 subjects (Audio)	Depression	KNN	79.27%	[23] 2018
Shannon entropy, spectral entropy + others	DEAP (32 channels)	32 subjects (Video)	Peace, anger, joy, depression	LSSVM	65.13%	[31] 2018
Conditional entropy (CEn) QSampEn	DEAP (32 channels)	32 subjects (Video)	calm and distress	SVM	80.31%	[32] 2020
Differential entropy	SEED (62 channels)	15 subjects (Video)	Positive and negative	LDA	68%	[33] 2019
	SEED (62 channels)	15 subjects (Video)	Positive and negative	MLP, CNN	83.7%	[55] 2022
Dynamic differential entropy (DDE)	SEED (62 channels)	15 subjects (Video)	Positive and negative	DDELGCN	81.56%	[56] 2022
*Multiscale entropy indices*						
Composite multiscale quadratic sample entropy (CMQSE), composite multiscale amplitude aware permutation entropy (CMAAPE)	DEAP (32 channels)	32 subjects (Video)	Valence and arousal	SVM, DT	86.35%	[34] 2019
Multi wavelet entropy	DEAP (32 channels)	32 subjects (Video)	valence, arousal, dominance, liking	SVM, FCM4	73.32%	[35] 2019
MSpEn6 + others	SEED (62 channels)	15 subjects (Video)	3 emotions (positive, neutral, and negative)	ARF	94.4%	[37] 2021
*Present study*						
Tsallis entropy (*q* = 2, 3, 4)	SEED (62 channels)	15 subjects (Video)	Positive, and Negative	KNN	*q* = 2 Avg Accuracy—71% Avg F_score_—0.69 Max Accuracy—79% Max F_score_—0.83	2023
					*q* = 3 Avg Accuracy—79% Avg F_score_—0.81 Max Accuracy—84% Max F_score_—0.87	
					*q* = 4 Avg Accuracy—71% Avg F_score_—0.68 Max Accuracy—80% Max F_score_—0.82	

Regularity in the EEG context is defined as the rate of the repetitiveness of specific patterns in the signal. Some of the widely used regularity-based entropy are approximate entropy and sample entropy. This entropy works on the probability of having repetitive patterns within the length of the signals selected. Studies using approximate and sample entropy gave accuracy between 73% and 90%.

Predictability defines the stability and the deterministic evolution of the nonstationary systems in time. Some of the entropies in this category are Shannon entropy (ShEn) and its generalization Renyi and Tsallis entropy and another variant of ShEn differential entropy. These entropy matrices are based on the probability distribution of the amplitude of the signal. Permutation entropy is also an example of it. Literature shows that the accuracies obtained from these matrices are between 65% and 82% [28–32].

Considering the multiscale nature of the EEG data set, multiscale entropy has been introduced. Multiscale entropy is computed by decomposing the signal into coarse-grained time series scales. It comprises of all the entropy stated above calculated in multi scales. Recognition accuracies from multiscale entropy matrices range between 73% and 94% [33–37].

### Feature Selection: Why Tsallis entropy?

The previous section discussed various entropy measures used in emotion recognition. Yet among several others, Tsallis entropy, a very potential generalized form of Shannon’s entropy remains unexplored in EEG-based emotion recognition. Tsallis entropy (TsEn) [38, 39] explores the nonextensive statistics of a system. It successfully describes systems having either multifractal space–time constraints, long-range interactions, or long-term memory effects [40]. Tsallis entropy incorporates a nonextensive parameter ‘*q*’ which acts as a zoom lens to study all systems varying from short- to long-range interactions.

### Mathematical formulation

Since 1948, Shannon’s entropy has been the fundamental and most widely used entropy to evaluate system complexity. Mathematically, Shannon’s Entropy is [15]

1 $${E}_{Sh}=-\sum_{i=1}^{N}{P}_{i}\,{\text{ln}}\,{P}_{i},$$

where *N* is the microscopic configuration of the system and ${P}_{i}$ is the probability of occurrence of the $i$ ^th^ configuration, and the sum of the probabilities should be unity, i.e., $\sum_{i}{P}_{i }=1$.

Limitation: Shannon’s entropy is valid for systems with short-range interaction and fails to comprehend systems with long-range interaction.

${E}_{Sh}$ is additive, ${E}_{Sh}\left(X\cup Y\right)= {E}_{Sh}\left(X\right)$ + ${E}_{Sh}\left(Y\right)$ Meaning systems X and Y are independent.

To overcome this limitation, a non-additive statistic was proposed [38, 39] by Tsallis therefore named Tsallis entropy. It is formulated as

2 $${E}_{ts}= \frac{1-\sum_{i=1}^{N}{P}_{i}^{q}}{q-1}$$

When $q\to 1$, ${E}_{ts}$ reduces to the definition of ${E}_{Sh}$ as:

$$\begin{aligned} ~E_{{ts}} = {\text{~}} & \frac{{1 - \mathop \sum \nolimits_{{i = 1}}^{N} P_{i}^{q} }}{{q - 1}} = \mathop \sum \limits_{{i = 1}}^{N} P_{i} \frac{{P_{i}^{{q - 1}} - 1}}{{1 - q}} \\ = & \mathop \sum \limits_{{i = 1}}^{N} P_{i} \frac{{{{\left( {q - 1} \right)\ln P_{i} }} - 1}}{{1 - q}} \\ ~ \approx & {\text{~}}\mathop \sum \limits_{{i = 1}}^{N} P_{i} \frac{{\left[ {1 + \left( {q - 1} \right)\ln P_{i} } \right] - 1}}{{1 - q}} \\ = & \mathop \sum \limits_{{i = 1}}^{N} P_{i} \ln P_{i} \\ \end{aligned}$$

As ${E}_{ts}$ follows nonextensive statistics and it undertakes the rule of pseudo additivity as

3 $${E}_{ts}\left(X\cup Y\right)= {E}_{ts}\left(X\right)+ {E}_{ts}\left(Y\right)+\left(1-q\right){E}_{ts}\left(X\right){E}_{ts}\left(Y\right)$$

In Eqs. (2) and (3), *q* is a parameter that measures the degree of non-extensivity [40]. A value of *q* = 1 corresponds to extensivity, that is, Shannon’s entropy. On the other hand, *q* < 1 corresponds to super-extensive, ${[E}_{ts}\left(X\cup Y\right)>{E}_{ts}\left(X\right)+ {E}_{ts}\left(Y\right)$ and *q* > 1 corresponds to subextensive [${E}_{ts}\left(X\cup Y\right)<{E}_{ts}\left(X\right)+ {E}_{ts}\left(Y\right)$] statistics.

As explained, Tsallis’ work presents a generalized form of Shannon’s entropy which can effectively describe systems or phenomena with long-range interaction. Primary electrical responses are generated from the cortical neurons [41]. After reaching a threshold value, these activation potential travels and reaches the brain scalp and is the recorded as EEG with respect to time and space. Therefore, EEG comes with inherent nonextensivity because of the long-range correlation that exists among billions of neurons [42]. These long rage interactions are the electrical information which are transmitted across different cortical areas and feedback loops composed of corticothalamic and thalamocortical networks [43]. Above argument suggests it is theoretically reasonable to replace existing entropy measures with Tsallis entropy to get a grip on the long-range effects of EEG. It is also sensible to consider EEG as a subextensive system (i.e., *q* > 1), since mutual information exists among different neuron clusters [44, 45].

### Research questions

As far as we know, this study is the first to propose Tsallis entropy as a feature (extracted from EEG) to study cross-subject emotions using SEED data sets. The present article explores the answers to the following queries:

- Are Tsallis entropy-based features reliable when attempting to extract complex information from EEG data sets to classify emotions?
- Does the classification performance depend on the entropic index “*q*,” also called the Tsallis parameter?
- Is Tsallis entropy as reliable for obtaining information from EEG data sets as Shannon entropy or other entropy indices? The study assesses the robustness of the feature vectors retrieved in terms of ‘accuracy’ and ‘*F*1 score’ performance metrices.

It also investigates the number of effective EEG electrodes, appropriate brain region, and advisable frequency range that could be preferred to study emotion identification in the future.

## Materials and methods

**Fig. 1:**
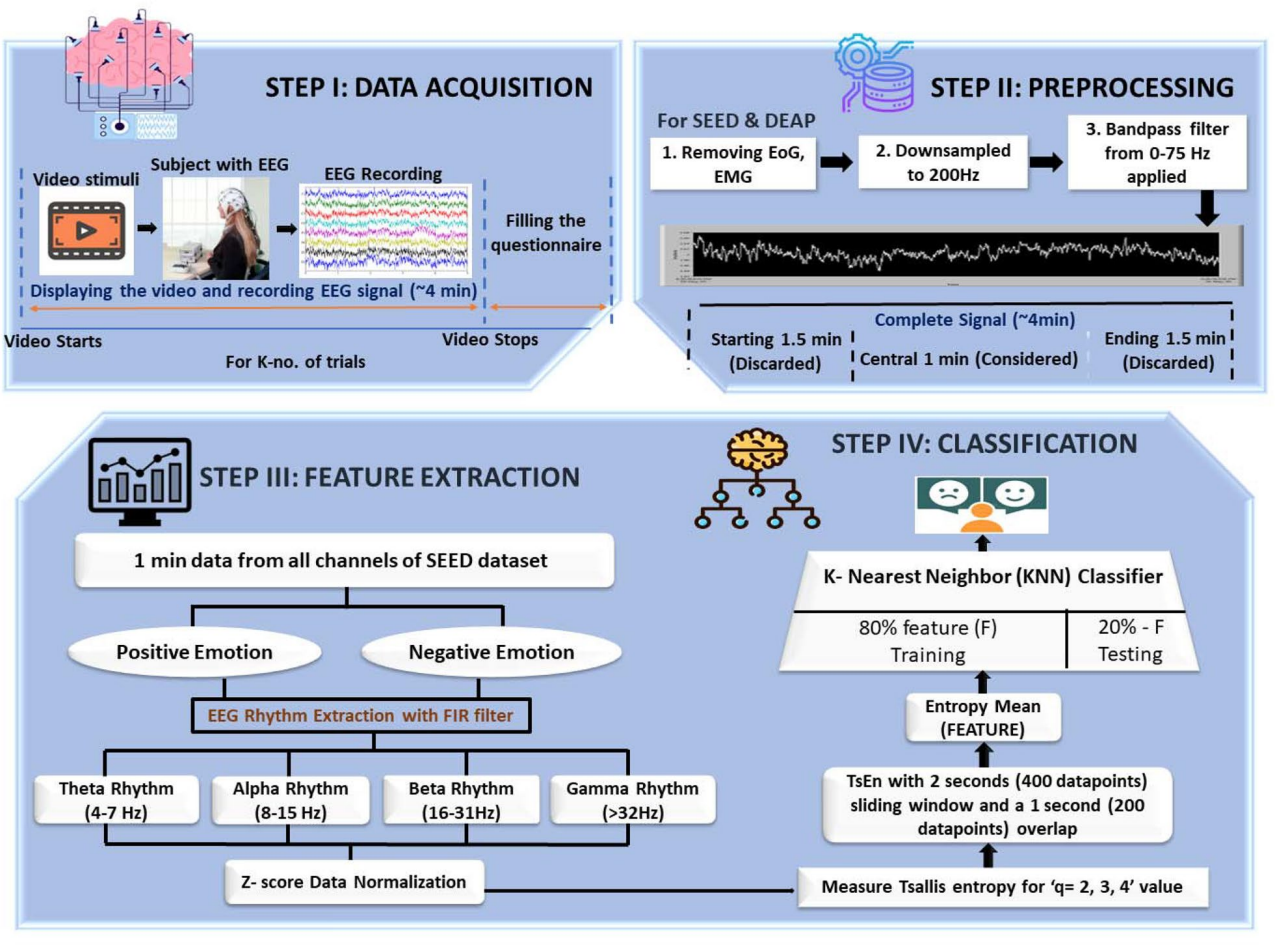
Illustration of proposed feature engineering (Tsallis entropy)-based human emotion recognition framework

The methodology diagram in Fig. 1 illustrates the essential phases of the research proposed. It consists of 4 steps each of which is explained in further subsections.

### Experimental data

We analyzed the publicly accessible data set SEED (SJTU emotion EEG data set) [46]. The SEED data set comprises of 62-channel EEG data which are collected from 15 test subjects (Seven males and eight females aged between 20 and 30 years), The experiment were repeated on the participants three times and the subjects’ emotions are induced using approximately four minutes long 15 film clips. The videos are organized in such a way that three emotions (positive, neutral, negative) classes can be examined with five corresponding film clips. In this study, only positive and negative data sets are used to evaluate feature’s performance for binary classification employed a one-minute-long data extracted from the middle part of each trial in the SEED.

### Data preprocessing

EEG data sets are high-dimensional neurophysiological signal which comprises redundant and noisy data. After data acquisition, electrooculogram (EOG) and electromyogram (EMG) artifacts and line interference were removed in the pre-processing step. This data was then downsampled to the sampling frequency of 200 Hz to eradicate the computational complexities in extracting the features. Then the one-minute-long signal was extracted from the central part of each SEED trial, which is the data of ~ 4 min duration.

This work also intends to study the emotion recognition in different frequency bands/rhythms of EEG rather than just the fundamental frequency, which is 0–75 Hz in our data case. Here, the FIR filter is used to extract different EEG rhythms, i.e., *θ* (4–7 Hz), *α* (8–15 Hz), *β* (16–31 Hz), and *γ* (32–55 Hz). Delta rhythm is not considered in this study, because it is mainly related to deep sleep activity. Before extracting features from these EEG rhythms, data were normalized using the *z*-score method, which for a random variable ‘*Y*’ with the mean ‘*Ῡ*’ and standard deviation ‘*δ*’ is stated as.

*Z* = (*Y*- *Ῡ*) / *δ*.

This process contributed to eliminating subject bias and generating more comparable features between subjects while preserving the variability of different channels. Then the required feature was extracted.

### Feature engineering

#### Tsallis entropy

**Fig. 2:**
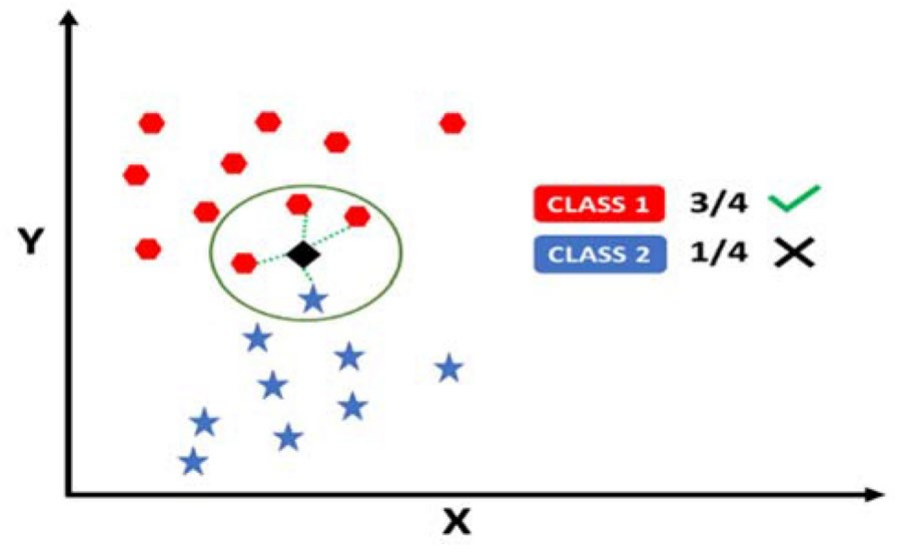
K-nearest neighbor classification model

Entropy, in general, depicts the unpredictability of any signal. The idea is to obtain a temporal variation of Tsallis entropy by incorporating a sliding time window in the input signal, then calculating the mean of the entropy obtained from the buffered signal. Suppose [*X(n): n* = 1,…, *L*] is the input signal. A sliding window *W* is designed such that the width of window ‘*w*’ is *w* ⩽ *L*, and the sliding step is *δ* ⩽ *w*. Above defined time-dependent Tsallis entropy in Eq. (2) is then computed in each sliding window where *Pi* is the probability. The probability defined here is the ratio of the number of *X*(*i*)-values of the sliding window and the total number of *X*(*i*)-values in the window. The mean and variance of this Tsallis entropy serve as the average local abnormality marker of the EEG signal to be analyzed and help differentiate the positive and negative emotions in different EEG rhythms (Fig. 2).

#### K-nearest neighbor classifier

K-nearest neighbor algorithm is the simplest of all machine learning algorithms. It is a non-parametric algorithm based on a supervised learning method. It classifies unknown data based on its neighboring data points, as shown in Fig. 2. The classification takes two steps; taking the assigned number of nearest neighbours first, then utilising the results of the first step to categorize the data point into a specific class:

4 $$Distance \left(x,y\right)=\sqrt{\sum\nolimits_{i}{\left({x}_{i}-{y}_{i}\right)}^{2}}$$

Hence, the performance of the KNN classifier depends on two factors i. ‘k’—the number of neighbors considered, ii. the distance to calculate the nearest data points. Various distance matrices could be used, such as ‘Cityblock,’ ‘Chebyshev,’ ‘Correlation,’ ‘Cosine,’ ‘Euclidean,’ ‘Hamming,’ ‘Jaccard,’ ‘Mahalanobis,’ ‘Minkowski,’ ‘Seuclidean,’ and ‘Spearman’. The parameter ‘k’ which is the number of neighbors could majorly depend on the size of the data considered. Upon computational optimization for the data considered in this work, we opted for ‘k = 10’—number of neighbors and ‘Euclidean’—distance defined in Eq. 4, to build our classifier.

#### DATA split and validation method

Any machine learning classifier is built with cross-validation partition methods. The cross-validation partition function is used to specify the type of cross-validation, and indexing is used to divide input data in training and testing sets, which depend on the research objectives. The three most basic strategies are ‘holdout,’ ‘k-fold,’ and ‘leave out. This work adapts the ‘hold out’ method for validation. In this method, a fraction of data specified as a scalar value in the range [0 1] is held for testing the model, and the rest of the data is used to train the classifier. We have used 0.2 holdouts in the present work, which means 80% of the features are used for training the k-NN classifier, and 20% is used for the method testing.

#### Performance evaluation metric

**Fig. 3:**
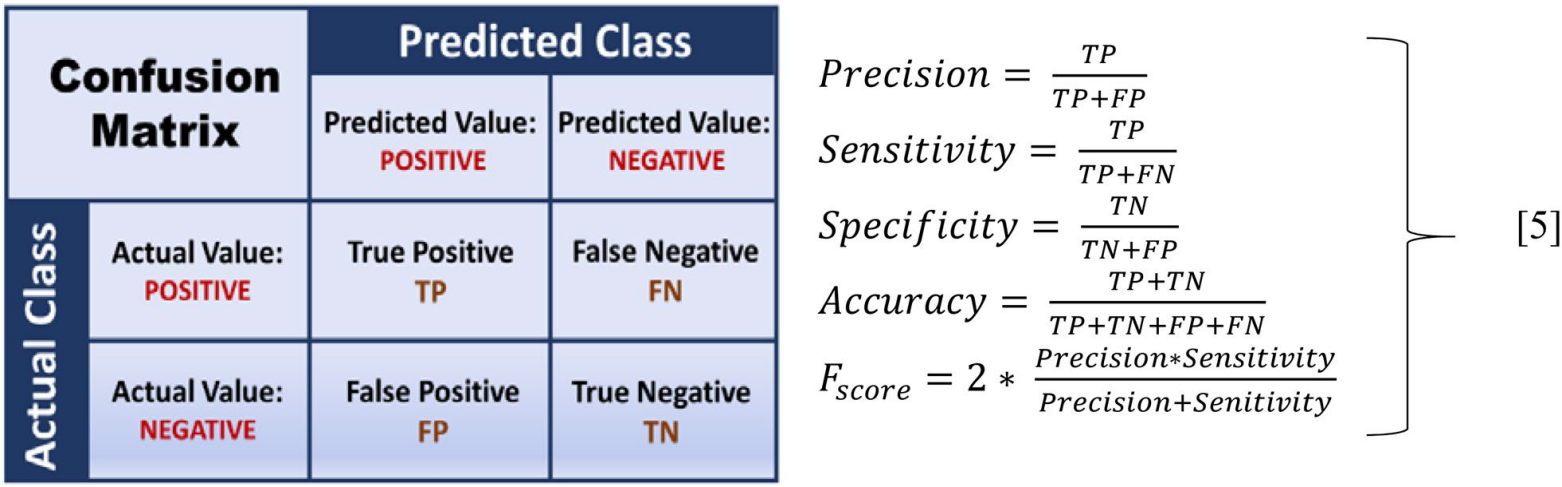
Confusion matrix illustration

Designed classifiers are commonly evaluated using a confusion matrix-based approach, as shown in Fig. 3, to ensure acceptable and reliable classification outcomes. Four classification metrics—accuracy, sensitivity, specificity, and *F*-score—can be obtained from the confusion matrix for performance comparison. For our study that undertakes a binary classification problem, the four metrics are defined by Eq. 5. The acronyms *TP*, *FP*, *TN*, and *FN*, stand for true positive, false positive, true negative, and false negative, respectively:

*TP* and *TN* are the number of true positive and negative samples correctly classified as positive and negative, respectively. Similarly, *FN* and *FP* are the number of true positive and negative samples misclassified as negative and positive, respectively. Accuracy and *F*-score is considered for the present study (Fig. 3).

## Results and discussion

**Fig. 4:**
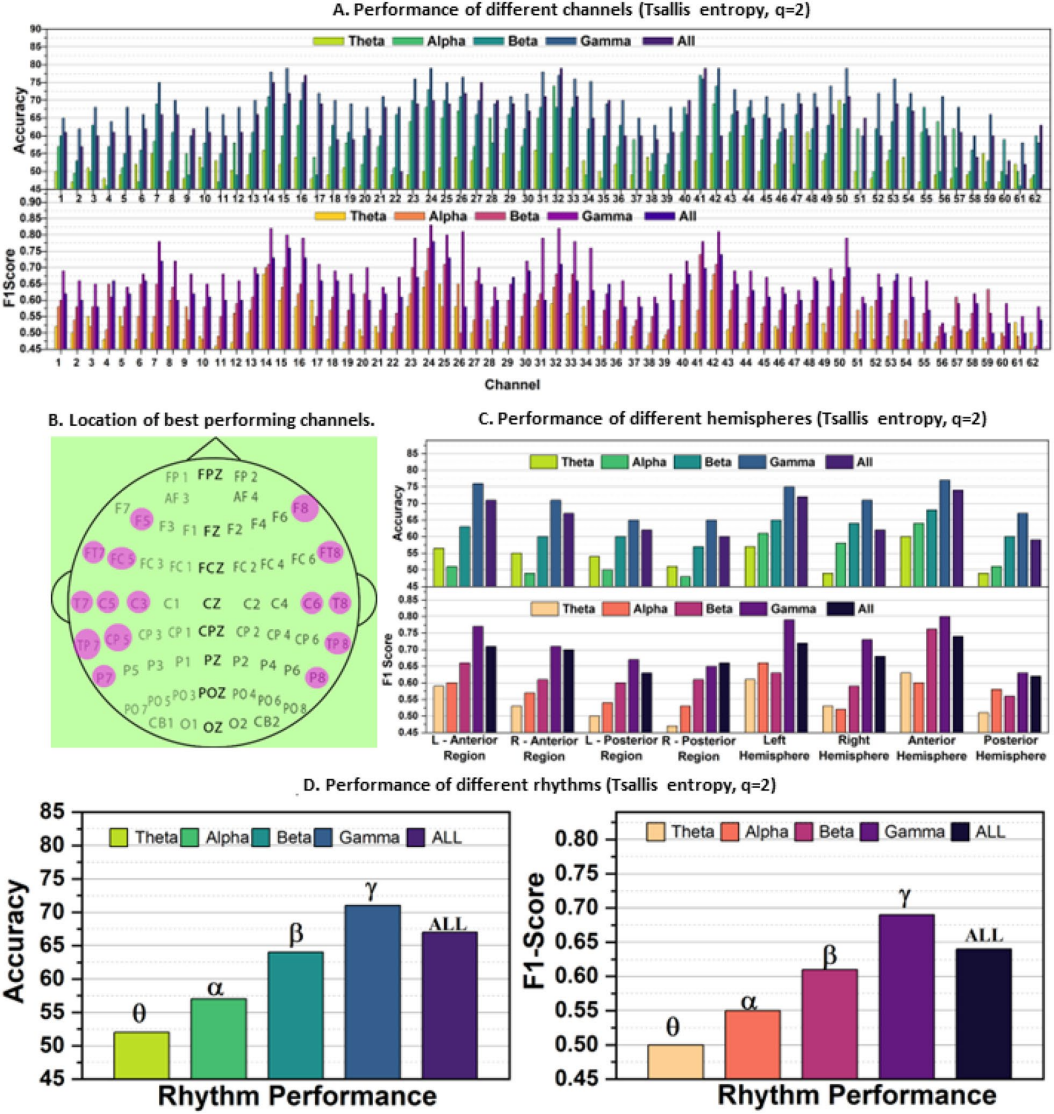
Subject independent emotion recognition performance **A** On different channels. **B** Brain topology indicating 1/4th of the total channels that performed the best. **C** of different brain hemispheres, **D** of different rhythms, all taking Tsallis entropy as feature for *q* = 2

**Fig. 5:**
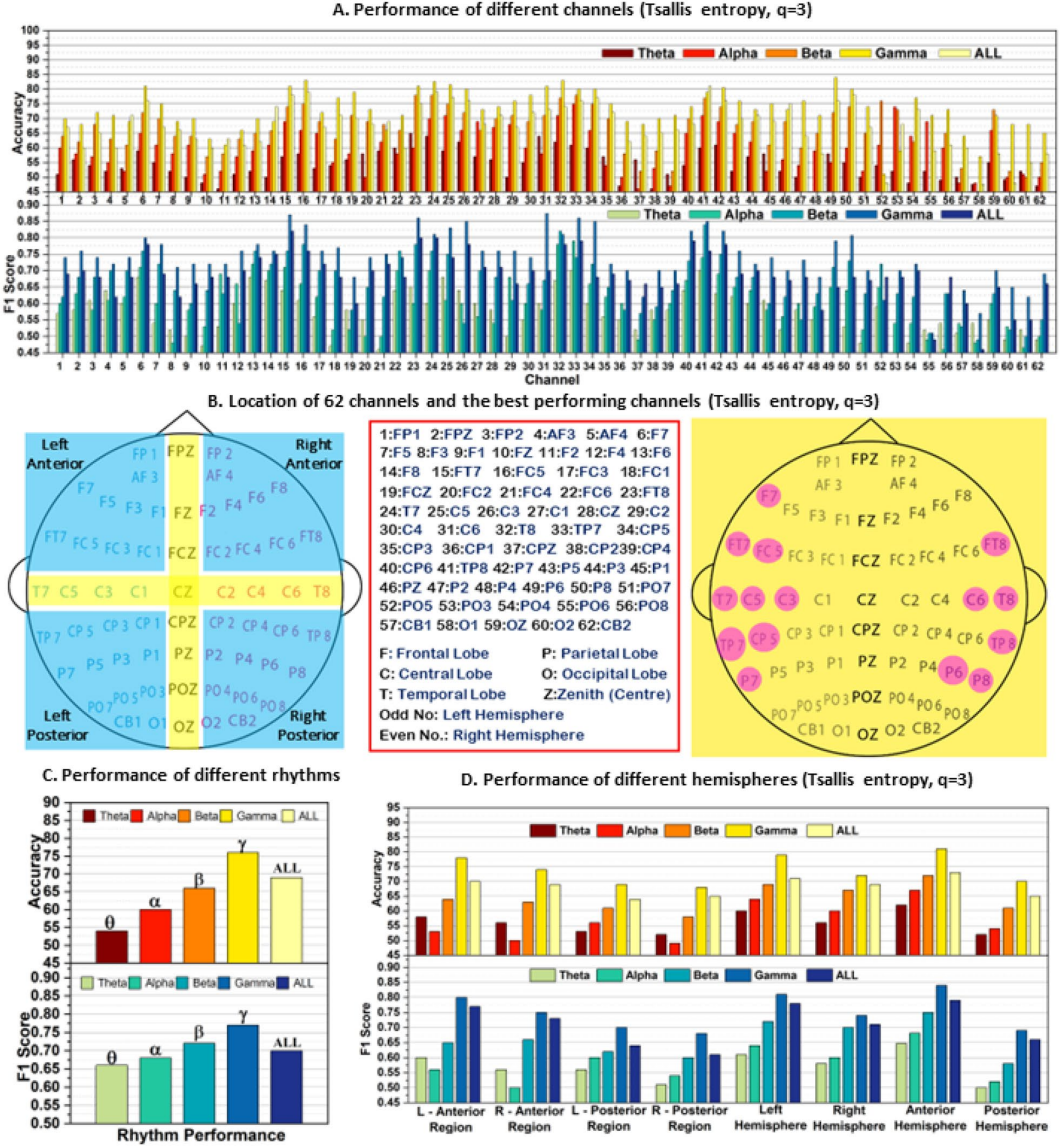
Subject independent emotion recognition performance **A** On different channels. **B** Brain topology describing channel location and nomenclature and indicating 1/4th of the total channels that performed the best. **C** Performance of different brain hemispheres, **D** Performance of different rhythms, all taking Tsallis entropy as feature for *q* = 3

**Fig. 6:**
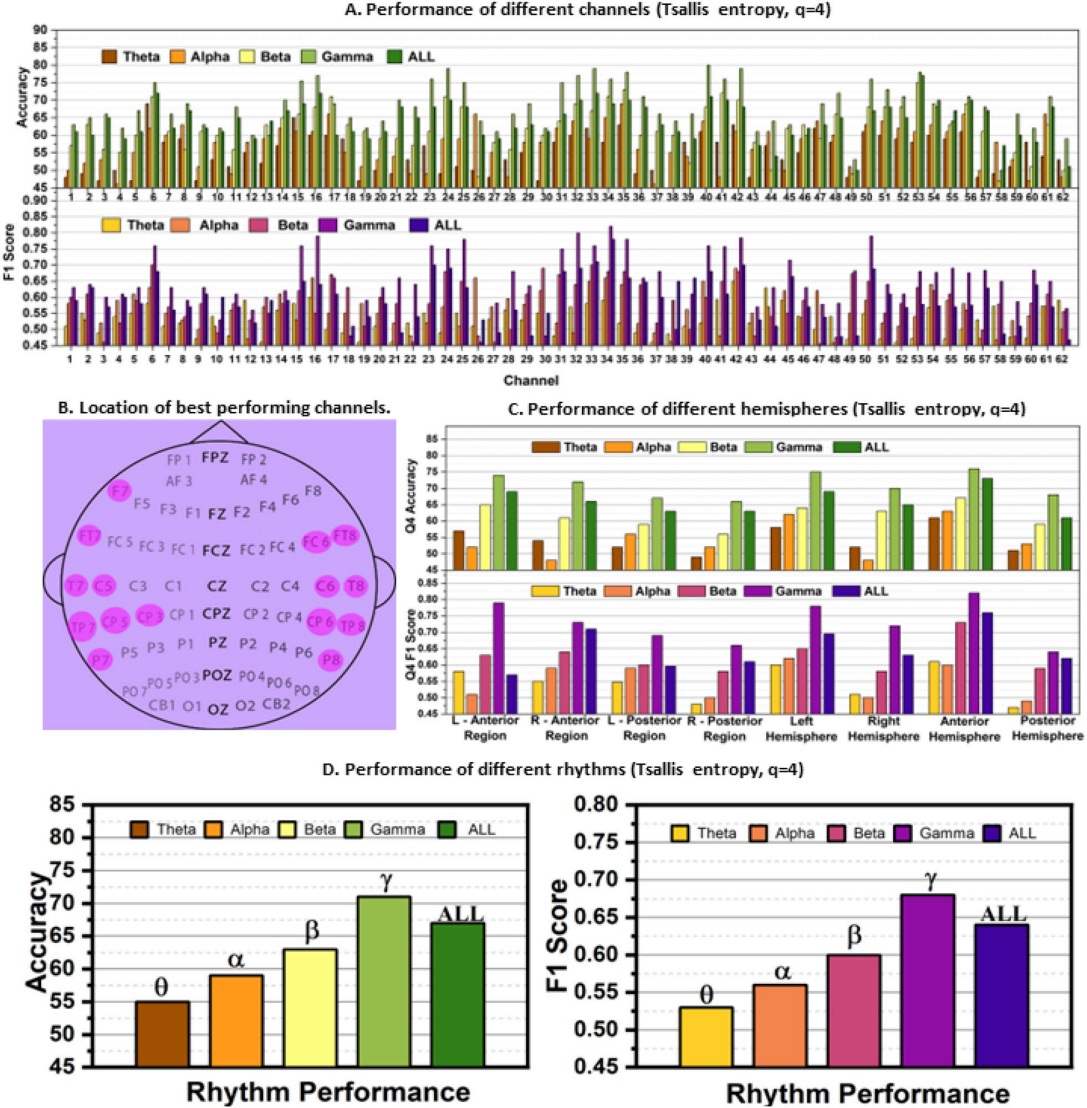
Subject independent emotion recognition performance **A** On different channels. **B** Brain topology indicating 1/4th of the total channels that performed the best. **C** of different brain hemispheres, **D** of different rhythms, all taking Tsallis entropy as feature for *q* = 4

All the computations explained above in the material and methods sections were carried out in MATLAB. Initially, the data was pre-processed, and the defined rhythms were extracted and normalized. Further, the Tsallis entropy feature for Tsallis parameter *q* = 2, 3, 4 was computed the classification of binary emotion was done through the KNN classifier. Finally, hold-out cross-validation was used by splitting each participant’s samples into an 80% training set and a 20% test set, keeping a roughly constant percentage of each class in each set relative to the original data. The classification process was run ten times to decrease the unpredictability induced by the data set’s random partition, and the average classification accuracy was calculated. As indicated, KNN machine learning algorithms were utilized for categorization work. A hyperparameter optimization approach was used to adjust the parameters of the classifiers. The study aims to find how well the Tsallis entropy performs in determining subject independent emotions. It considers the perspectives of different channel locations, all the brain regions, and certain rhythms. The results are shown in Figs. 4, 5, 6.

*Overall Evaluation* In this study, the performance of 62 channels from the SEED data set was evaluated for Tsallis parameters 2, 3, and 4, with accuracy and *F*-score presented in Figs. 4A, 5A, and 6A. The top-performing channels (one-fourth of the total) were marked on the brain diagram in Figs. 4B, 5B, and 6B. The results show that electrodes from the temporal lobe on both sides of the brain exhibited superior performance in differentiating positive and negative emotions, irrespective of the subjects.

Furthermore, the study explored the performance of different EEG rhythms. Figures 4D, 5C, and 6D reveal that the gamma rhythm (31–55 Hz frequency range) achieved the best performance, followed by the beta rhythm. All frequency bands showed comparable results as well. This indicates that higher frequency rhythms, specifically beta (*β*) and gamma (*γ*), play a more critical role in emotion study compared to lower frequency rhythms like theta (*θ*) and alpha (*α*).

In the study, the performance of different brain regions was analyzed separately and presented in Figs. 4C, 5D, and 6C. Figure 6B shows the partitioning of the brain regions into eight distinct regions of interest. When evaluating the four quadrants, namely left anterior, right anterior, left posterior, and right posterior regions, electrodes from the intersecting regions were excluded from consideration. The analysis of lower gamma bands from the left and right profiles of the brain revealed that the left anterior and posterior regions exhibited superior performance compared to the right anterior and posterior regions, respectively.

Furthermore, the left hemispheres consistently outperformed the right hemispheres across all frequency bands and cases. Moreover, when comparing the results between posterior and anterior regions, the features from the anterior hemispheres consistently demonstrated better classification performance across all frequency bands.

### Comparative study of Tsallis parameter q-based performance

The study acknowledges the significance of different values of *q* in entropy computation for EEG research, as extensively discussed in the literature [47–50]. The proposed method's performance is compared based on accuracy and *F*-score metrics. While accuracy represents the proportion of correct predictions made by the classification model, it can be misleading when used as the sole criterion for assessing performance. This is because accuracy depends on correctly predicted positive and negative classes, and higher accuracy with a higher number of incorrect predictions may indicate poor performance. To address this limitation, the study incorporates the *F*-score matrix for performance evaluation. The *F*-score considers the impact of false negatives and false positives, making it particularly relevant when studying negative emotions and providing a more comprehensive assessment of the proposed method’s performance. By considering both accuracy and *F*-score, the study aims to provide a more robust evaluation of the classification method's effectiveness in emotion differentiation using EEG data.

The findings from Figs. 4, 5, and 6 highlight the superior performance of the gamma band in comparison to other frequency bands. Further analysis focuses on the numbers obtained from the gamma band's performance matrices. For ‘*q* = 2’, the maximum accuracy–*F*-score pair is 79%–0.83, and the best average accuracy–*F*-score pair is 71%–0.69. For ‘*q* = 3’, the maximum accuracy–*F*-score pair is 84%–0.87, and the best average accuracy–*F*-score pair is 79%–0.81. For ‘*q* = 4’, the maximum accuracy–*F*-score pair is 80%–0.82, and the best average accuracy–*F*-score pair is 71%–0.68. The study reveals that ‘*q* = 3’ outperforms the other values of *q*, although the differences between ‘*q* = 2’ and ‘*q* = 3’ are not substantial. The presence of relatively low *F*-scores compared to accuracy for ‘*q* = 2’ and ‘*q* = 4’ indicates higher false negative and false positive rates in the predictions, emphasizing the need for cautious interpretation of results based solely on accuracy. On the other hand, ‘*q* = 3’ exhibits competitive accuracy and *F*-score, making it an excellent choice for further study. This underscores the importance of carefully selecting the nonextensive parameter *q* and the need for additional research to optimize its value for different studies.

A comparative analysis of Figs. 4B, 5B, and 6B identifies common electrode positions that consistently performed well across all cases of *q*. These electrodes include FT7, FT8, T7, C5, C6, T8, TP7, CP5, TP8, P7, and P8. These positions can be considered for further emotion studies using the SEED data set.

## Conclusion

In this work, we have explored a novel emotion recognition method based on Tsallis entropy and the KNN Classifier, using the SEED (EEG) data set. The study successfully addressed the research questions outlined in Sect. 1.6.

The proposed method achieved the best average accuracy of 79% and a maximum accuracy of 84%, accompanied by *F*-scores of 0.81 and 0.87 for *q* = 3. This finding confirms that Tsallis entropy is effective in assessing chaotic situations in EEG signals, encompassing inconsistencies, complexities, and unpredictability. As a result, Tsallis entropy holds significant relevance in emotion recognition tasks.

Our study further found that the model's performance is influenced by the Tsallis parameter *q*, although the variation is not deemed significant, as discussed in response to the second research question. However, the paper does not delve into the detailed explanation for this variation, considering it beyond the scope of the current study. Nevertheless, a comparison of the present study's results with previous works presented in Table 1 demonstrates that Tsallis entropy indeed yields competitive performance compared to various state-of-the-art techniques, thereby addressing the third research question. The study highlights the crucial role of the gamma rhythm in generating efficient features that lead to higher performance in emotion recognition, aligning with previous literature. This reaffirms the significance of the gamma rhythm in EEG-based emotion studies. The proposed method's advantage lies in the simplicity of Tsallis entropy, which possesses low computational complexity and nonlinearity. The TsEn features effectively extract hidden complexities in EEG signals, resulting in improved accuracy.

However, the study also acknowledges several limitations. One major limitation is the necessity to optimize the entropy index ‘*q*’ for each specific research task, which can be a challenging and time-consuming process. In addition, the classification accuracy achieved by the proposed method is not very high, indicating the need for further modifications and improvements. In future work, the authors plan to enhance the model’s performance by integrating the extracted features with deep learning models. This could potentially lead to increased accuracy and more robust emotion recognition. The proposed method will also be evaluated on other emotion data sets to ensure its reliability and generalizability across different data sets and scenarios. This will provide a comprehensive assessment of the method’s effectiveness in real-world emotion recognition applications.

## Data Availability

The SEED database used in the present analysis can be found at http://bcmi.sjtu.edu.cn/home/seed/seed.html.
